# Letter to the Editor: A response to Hruska’s case study on molecular breast imaging and the need for true tissue quantification

**DOI:** 10.1186/s13058-019-1103-6

**Published:** 2019-01-29

**Authors:** Richard M. Fleming, Matthew R. Fleming, Tapan K. Chaudhuri, William C. Dooley, Andrew McKusick

**Affiliations:** 1 FHHI-Omnific Imaging-Camelot, Los Angeles, CA 90245 USA; 2 Eastern Virginia Medical School, Norfolk, VA USA; 3 Oklahoma University Health Science Center, Oklahoma City, OK USA; 4 Sebec Consulting & Media, Charlotte, NC USA

**Keywords:** FMTVDM©℗; B.E.S.T. Imaging©℗, Breast cancer, Breast inflammation, Theranostics, Quantification, AI, Nuclear camera quantitative calibration, Patent protected

We applaud the efforts by Hruska et al. [1] to quantify differences in tissue using molecular breast imaging (MBI) and background parenchymal uptake (BPU); we have discussed the use of such previously [2]. The approach while commendable did not provide diagnostically useful information to differentiate tissue types. This approach, like the utilization of standardized uptake value (SUV), compares differences in background with tissue [3]. As we have already discussed [2, 4, 5] in the literature, this approach is an incorrect model, due to (1) the critical lack of standardization and calibration of nuclear cameras including both SPECT/Planar and PET; (2) the utilization of ratios which are not absolute values and therefore cannot be used to differentiate tissue based upon those issues, issues which are critical to the understanding of tissue differences; and (3) the inability to truly “measure” transitional changes in tissue, which would allow for the determination of actual treatment response on a per patient basis, saving time, money, and lives.
